# Fully Quantized Training vs. Post-Training Quantization for a Small Hyperspectral Transformer Model for Pixel-Level Foreign Plastic Object Classification

**DOI:** 10.3390/s26175531

**Published:** 2026-08-31

**Authors:** Zirak Khan, Seung-Chul Yoon, Suchendra M. Bhandarkar

**Affiliations:** 1 School of Computing, University of Georgia, Athens, GA 30602, USA; zirak.khan@uga.edu (Z.K.); suchi@uga.edu (S.M.B.); 2 Quality and Safety Research Unit, U.S. National Poultry Research Center, U.S. Department of Agriculture—Agricultural Research Service, Athens, GA 30605, USA

**Keywords:** hyperspectral imaging, fully quantized training, post-training quantization, model compression, fqt, ptq, foreign object detection, foreign plastic objects, NVIDIA Blackwell, NVFP4, FP8, transformer, food safety, sensor technology

## Abstract

Low-precision floating-point computation has become central to efficient artificial intelligence, yet its behavior for compact transformer-based hyperspectral imaging (HSI) models remains underexplored. In this work, we present a controlled comparative study of fully quantized training (FQT) and post-training quantization (PTQ) for pixel-wise foreign plastic object (FPO) classification in poultry hyperspectral data. Using a fixed state-of-the-art spatial–spectral transformer backbone, a common mixed-precision strategy, and identical training and inference protocols, we evaluate FP32, FP16, BF16, FP8, and NVFP4 across predictive performance, model compression, training efficiency, and inference efficiency. The results show that mixed-precision FQT remains highly robust across the tested precision spectrum, with all reduced-precision configurations staying within 0.63 percentage points of the FP32 baseline in overall accuracy while consistently outperforming PTQ at matched precisions. Across the evaluated formats, BF16 provides the closest accuracy to FP32, whereas FP8 offers a particularly favorable balance between accuracy preservation and reduced precision, while model compression increases progressively to 3.69× under NVFP4. The computational benefits, however, are strongly workload dependent. Native FP8/FP4 hardware support does not automatically improve training throughput for this compact model at moderate workloads, and the larger training batches required to better utilize low-precision hardware can degrade predictive performance. In contrast, large-batch inference can effectively exploit FP8 and NVFP4 without affecting predictive accuracy. An ablation study further shows that selective retention of numerically sensitive modules in FP32 is essential for stable ultra-low-precision operation. Overall, the findings demonstrate that low precision is a viable but workload-dependent design choice for compact HSI transformers, with FQT providing greater accuracy robustness than PTQ and FP8, offering a favorable overall accuracy–efficiency trade-off.

## 1. Introduction

Hyperspectral imaging (HSI) captures both dense spectral information, by recording hundreds of narrowly spaced contiguous bands for each pixel, and high-resolution spatial information [1]. HSI enables not only non-destructive analysis of material properties, but also detailed spatial feature extraction [2]. As a result, HSI has become a key technology in high-impact applications ranging from food safety and quality control to remote-sensing-based environmental monitoring and land-cover mapping [3,4,5]. One particularly important application in food safety is foreign object detection (FOD) and classification, which seeks to identify small (≈5  mm) non-biological foreign objects (FOs), especially low-density foreign plastic objects (FPOs), in food products, objects that are often difficult to detect using other imaging modalities such as X-ray or conventional RGB vision [3,6,7]. These FOs in food production lines pose severe health risks to consumers, but can also result in costly product recalls and brand damage [8]. Efficient detection and removal of such contaminants are therefore essential for maintaining food-safety standards and ensuring regulatory compliance. More recently, deep learning (DL) approaches, particularly HSI-based and transformer-based methods, have increasingly outperformed traditional methods that primarily rely on spectral analysis in a variety of hyperspectral computer vision tasks across both remote sensing and food inspection, owing to their ability to learn complex spatial–spectral representations [4,5]. However, the same properties that make HSI powerful, high spectral dimensionality and fine spatial resolution, also create significant efficiency bottlenecks. Modern deep HSI classifiers must process large spatial–spectral tensors, and transformer-based architectures further amplify this cost by modeling complex spatial and spectral interactions through computationally expensive attention mechanisms and large general matrix multiplications (GEMMs), making them both memory- and compute-intensive [9]. These efficiency constraints hinder the practical deployment of transformer-based HSI models, particularly in throughput-constrained environments such as industrial food inspection lines [10].

Low-precision numerical representations and arithmetic can significantly improve the efficiency of both training and inference in deep learning models. Reducing numerical bit-width with low-precision operations decreases memory footprint and can lower the cost of computation and data movement, thereby improving overall efficiency [11,12,13]. Quantization provides the mechanism for achieving this by mapping high-precision values to lower-bit representations. It has become an effective strategy for reducing memory requirements, computational costs, and improving overall efficiency, particularly in large pretrained models. These advantages motivated the adoption of 16-bit floating-point formats, such as Google’s Brain Floating Point 16-bit (BF16) [14], as a standard alternative to the full-precision floating-point format (FP32) in the training of early large language models (LLMs). BF16 not only reduces memory usage and improves efficiency but also enhances numerical robustness by preserving an FP32-like dynamic range, with 8 bits for the exponent [15]. In recent years, 8-bit floating-point (FP8) formats have also been introduced, and recent studies suggest that FP8 is both feasible and effective for training and inference in LLMs, while offering additional efficiency gains [16,17,18,19]. Consequently, FP8 is rapidly emerging as a new standard for large-scale model training in both natural language processing and computer vision [17,18]. More recently, NVIDIA introduced the Blackwell GPU architecture, representing a major milestone as the first GPU to natively support 4-bit floating-point (FP4) operations such as matrix multiplication via specialized tensor cores [20]. This development has pushed the precision frontier even further, and preliminary studies have proposed FP4 formats with different scaling-factor strategies and block sizes that show promising performance and energy-efficiency gains for LLM training while maintaining model accuracy and training stability [21]. Beyond training, these low-precision formats have also been widely studied for inference efficiency, and 8-bit and 4-bit numerical formats have increasingly become standard choices for inference in large pretrained models. However, although such formats have been actively explored for LLM training, especially FP8 and FP4, it remains unclear whether aggressive quantization strategies that are effective for large pretrained vision or language models transfer directly to much smaller transformer-based HSI models [18,22]. HSI inputs consist of continuous, high-dimensional spectral signatures, where discriminative information may be encoded in subtle magnitude differences across correlated bands; as a result, quantization noise may interact with the spectral feature space in domain-specific ways. Moreover, smaller models may derive limited benefit from specialized hardware accelerators such as Blackwell tensor cores, which natively support FP4 and FP8 matrix multiplication and typically realize their full advantage in the large-scale GEMMs characteristic of massive-model pretraining and inference.

In practice, model quantization approaches are categorized into fully quantized training (FQT), post-training quantization (PTQ), and quantization-aware training (QAT). The latter two are primarily inference-oriented. In PTQ, a pretrained model, typically trained in full precision, is converted to a lower precision without further training to enable more efficient inference [13,23,24]. In QAT, by contrast, the model is trained using higher-precision arithmetic for parameter updates, while quantization effects are simulated during training through fake-quantized weights and activations so that the model learns to remain robust under low-precision inference, thereby preserving accuracy more effectively than PTQ [25]. FQT differs from both PTQ and QAT in that it is not only intended to preserve robustness and accuracy under quantization but is also explicitly oriented toward training efficiency. In FQT, the model is trained predominantly in the target low precision throughout the training process; that is, weights, activations, and gradient computations are all performed in low-bit formats, enabling efficiency gains during training itself [21]. PTQ has been shown to be a practical and straightforward strategy for deploying low-precision inference when retraining is infeasible or computational resources are limited, as is often the case for large pretrained models [13,26]. PTQ typically relies on calibration statistics to estimate dynamic ranges, particularly for activations. Although effective at moderate precisions, PTQ can suffer performance degradation under more aggressive quantization levels and in models that are sensitive to activation outliers [23,27,28]. For large pretrained transformer models, specialized PTQ techniques, largely developed in the language-model literature, have further improved quantization parameter optimization for effective deployment [29,30]. QAT is typically used as a complementary approach to PTQ, yielding better accuracy preservation during inference because the model learns to compensate for quantization-induced errors during training. However, QAT requires access to the training recipe as well as additional retraining time and computational cost, making it more suitable when high accuracy must be maintained and retraining remains feasible. Unlike FQT, however, it does not provide gains in training efficiency. More recently, FQT has emerged as an effective approach for reducing training cost while also demonstrating stable convergence and numerical robustness at low precision compared with higher-precision training. Recent studies show that low-precision training can succeed when supported by appropriate scaling strategies and block-size design choices [17,21,31]. In addition, prior work has demonstrated the feasibility of FP8 and FP4 FQT on Blackwell GPUs by leveraging tensor cores for large-model training [17,21,32]. Beyond the question of when quantization is applied, an equally important consideration is how it is applied. Quantization does not necessarily require a uniform precision across all layers or operations of a neural network. Mixed-precision quantization assigns different bit-widths to different layers or computations according to their sensitivity to quantization. In doing so, it seeks to balance model size, computational efficiency, and predictive performance by allocating higher precision to more sensitive components and lower precision to less sensitive ones [33].

Despite the growing interest in low-precision computing and its demonstrated effectiveness for accelerating training and inference in large foundation models, a critical gap remains in understanding how aggressive quantization affects transformer models operating on high-dimensional hyperspectral data. Existing HSI studies predominantly rely on FP32 or FP16 training, while the comparatively limited use of quantization in HSI has largely focused on inference-oriented PTQ at moderate precisions. In contrast, emerging floating-point formats such as FP8 and FP4 have been investigated primarily for large-scale language and vision models, and their suitability for compact HSI transformers remains largely unexplored. Consequently, there is still no systematic understanding of whether such models can be reliably trained and deployed across progressively reduced floating-point precisions, or whether FQT provides an advantage over PTQ in preserving predictive performance. Moreover, the lack of a controlled evaluation across FP32, FP16, BF16, FP8, and FP4 limits the development of practical guidelines for selecting precision configurations in resource-constrained HSI applications. In this paper, we investigate mixed-precision quantization as a practical strategy for enabling efficient transformer-based HSI classifier training and inference without sacrificing predictive accuracy. Using a compact transformer for foreign plastic object classification in hyperspectral poultry images as a representative case study, we isolate numerical precision as the primary experimental variable by holding the architecture, optimizer, data pipeline, training protocol, and inference procedure constant while varying only the computation precision. We further distinguish between training-phase behavior under fully quantized training (FQT) and inference-phase behavior under both FQT and PTQ, providing a comprehensive analysis of the trade-offs between numerical precision, model accuracy, and computational efficiency. Our study not only characterizes the robustness limits of low-precision training but also identifies mixed-precision configurations that maintain accuracy even under aggressive quantization, providing practical guidance for efficient deployment of compact HSI transformers. Our contributions are fourfold:

1. **Accuracy-preserving mixed-precision strategies.** We identify mixed-precision configurations that retain near-full-precision accuracy under aggressive 8-bit and 4-bit quantization.
2. **End-to-end training-phase comparison across the precision spectrum for mixed-precision FQT.** We provide a controlled evaluation of transformer-based pixel-level FPO classification under FP32, FP16, BF16, FP8, and FP4, and distinguish the regimes in which FQT remains stable from those in which it leads to accuracy degradation.
3. **Joint inference accuracy–efficiency characterization of both FQT and PTQ.** Beyond headline OA, we report class-balanced and agreement-based metrics together with detailed training and inference efficiency measurements, enabling an accuracy–efficiency frontier analysis rather than a single-point comparison.
4. **Deployment-oriented guidance.** By explicitly comparing FQT and PTQ at inference under the same target precisions, we identify when PTQ is sufficient for rapid deployment and when FQT is justified to preserve accuracy under aggressive quantization.

## 2. Preliminaries

According to the IEEE 754 standard [34], a binary floating-point number consists of three components: a 1-bit sign (*S*), exponent bits (*E*), and mantissa (fraction) bits (*M*). In the machine learning literature, this representation is often denoted as *ExMy*, where *x* and *y* specify the number of exponent and mantissa bits, respectively. For instance, FP16 is represented as E5M10, whereas BF16 uses E8M7. FP8 typically comes in two variants, E4M3 and E5M2. In contrast, FP4 uses the E2M1 format, using two exponent bits and one mantissa bit for its 4-bit floating-point representation [35]. Unlike integer (INT) quantization, floating-point (FP) quantization is characterized by nonuniform quantization intervals and a wider dynamic range. To quantize a high-precision tensor such as FP32 into lower-precision FP formats, including FP16, BF16, FP8, and FP4, and to dequantize it accordingly, we formulate a unified quantization–dequantization framework in which each FP precision paradigm is treated as a special boundary case determined by its scaling-factor mechanism.

The generalized quantization of a tensor *X*, which may represent weights, activations, or gradients, into a low-precision tensor $X^{\left(q\right)}$ is defined as

(1) $$X^{\left(q\right)}=Q_{F}\left(\frac{X}{\sigma }\right)$$

where *X* denotes the full-precision input tensor, σ is the corresponding generalized scaling factor, and $Q_{F}\left(\cdot \right)$ is the projection operator that maps a full-precision value to the nearest discrete value in the finite set of representable values of a target low-precision format F (e.g., $F_{BF16},F_{FP16},F_{FP8},F_{FP4}$), thereby inherently encompassing operations such as rounding (e.g., round-to-nearest). The resulting quantized tensor satisfies $X^{\left(q\right)}\in F$ and is encoded in the target low-precision format.

To reconstruct the approximate full-precision tensor $\hat{X}$, the scaling factor is reapplied in dequantization as follows:

(2) $$\hat{X}=X^{\left(q\right)}\cdot \sigma$$

### 2.1. Defining the Scaling Factor per Precision

With the generalized projection framework established, the different low-precision paradigms can now be distinguished solely by the way the scaling factor σ is defined. Let $M_{F}=\max\left(F\right)$ denote the maximum representable absolute value of the target low-precision format.

#### 2.1.1. 16-Bit Unscaled Formats (FP16, BF16)

For formats with sufficient native dynamic range—or that share the same exponent size *E* as FP32—no explicit scaling is required. In this case, the generalized equation reduces to a pure projection with σ=1.

#### 2.1.2. FP8 Quantization (Per-Tensor Scaling)

FP8 formats (e.g., E4M3 and E5M2) commonly use a single scaling factor broadcast across the entire tensor, which represents a relatively coarse-grained per-tensor scaling strategy for mapping the tensor distribution safely into the dynamic range of the target format. Finer-grained alternatives also exist, such as block-level or microscaling-based approaches that assign separate scaling factors to groups of values; however, in our implementation through Transformer Engine on the laptop Blackwell GPU, FP8 was employed using the tensor-wise scaling formulation. FP8 tensors in the forward pass use the E4M3 format, whereas FP8 tensors in the backward pass use E5M2 because gradients require a greater dynamic range for stable training. The scaling factor is obtained using the widely adopted *absmax* method [18,36], computed from the maximum absolute magnitude of the tensor:

(3) $$\sigma =\frac{\max(|X|)}{M_{FP8}}$$

where max(|X|) denotes the maximum absolute value of the entire tensor *X*, and $M_{FP8}$ is the maximum representable value of the selected FP8 format.

#### 2.1.3. FP4 Quantization (NVFP4)

For ultra-low-precision formats such as FP4, the tensor is partitioned into contiguous sub-blocks to preserve local activation outliers better and maintain structural fidelity. In this work, we adopt the NVFP4 format, following recent low-precision training studies in the LLM literature [21], while noting that other FP4-style microscaling formats also exist and may differ in their block sizes and scaling strategies. Under this formulation, the scaling factor is defined as

(4) $$\sigma =s_{tensor}\cdot s_{block}$$

where $s_{tensor}$ is the scaling factor computed for the full tensor and stored in FP32, and $s_{block}$ is the local block-level scaling factor for each block of 16 consecutive elements, and each $s_{block}$ is stored in FP8 format to have better range [20]. Both of these scaling factors are computed using the same absmax method defined earlier.

### 2.2. Mixed-Precision Strategy for FQT and PTQ

Following the conceptual overview provided in the Introduction, this section formalizes the common mixed-precision framework and the technical distinctions between FQT and PTQ, with particular emphasis on their training, calibration, and scaling procedures. Quantization does not require assigning a uniform precision to every layer and operation of a neural network. Instead, mixed-precision quantization assigns different bit-widths to different computations according to their sensitivity to precision reduction. This design balances the trade-off among model size, computational efficiency, and predictive performance by preserving higher precision in numerically sensitive components while applying lower precision where greater compression and acceleration can be achieved with minimal loss. In this study, both FQT and PTQ follow the same mixed-precision strategy. As a result, the comparison between the two paradigms isolates differences arising from *when* quantization is introduced and *how* the scaling statistics are obtained, rather than from differences in precision allocation across the network.

#### 2.2.1. FQT

FQT integrates quantization directly into the training process. Both the forward and backward passes are performed predominantly in low-precision arithmetic, including weights, activations, and gradients, while higher precision is retained only where necessary to ensure numerical stability. Within the common mixed-precision framework adopted in this work, FQT is intended not only to improve training efficiency but also to reduce the accuracy degradation that can arise from applying quantization only after training. Because quantization effects are present throughout optimization, the model can adapt to quantization noise during learning, which often leads to better accuracy preservation, particularly at aggressive low precisions. In FQT, the ranges $\left(X_{min},X_{max}\right)$ are tracked during training; consequently, at inference time, these training-time statistics are reused to compute the scaling factor σ, eliminating the need for a separate calibration dataset.

#### 2.2.2. PTQ

PTQ, by contrast, is designed specifically for inference-time efficiency. Under the same mixed-precision strategy used for FQT, a pretrained full-precision model is converted to a lower precision after training, without any additional optimization, so that low-precision computation can be used during deployment. The process begins by determining the ranges $\left(X_{min},X_{max}\right)$ of model parameters and activations. Whereas the parameter ranges are directly available from the pretrained model, the activation ranges must be estimated using a representative calibration dataset. The scaling factor σ is then computed from these ranges and the target numerical format, following the scaling formulations described above. PTQ is attractive because of its simplicity and low overhead, making it suitable when retraining is impractical or computational resources are limited. However, because the model does not adapt to quantization noise during learning, PTQ can incur greater performance degradation, especially in smaller models that are highly sensitive to precision loss.

## 3. Experimental Setup

### 3.1. Datasets and Task

The experiments in this study are conducted on the same in-house *food-safety* hyperspectral dataset previously described in detail in [37] and collected for FPO analysis on poultry. Data acquisition was performed using a pushbroom line-scanning system (Micro-Hyperspec Extended VNIR R640, Headwall Photonics, Bolton, MA, USA) equipped with an InGaAs sensor, a 25 mm objective lens, and tungsten-halogen illumination. During acquisition, the fillet samples were placed on a computer-controlled moving stage that translated beneath the stationary hyperspectral sensor head at a lens-to-sample holding plate distance of 36 cm, while the camera exposure time was set to 70 ms. Although the sensor covers the 600–1700 nm range, our analysis is limited to the common industrial InGaAs near-infrared (NIR) range of 1000–1700 nm. Each hyperspectral cube has spatial dimensions of 638×1068 and contains 268 wavelength bands before band selection; after restricting the spectrum to 1000–1700 nm, each pixel is represented by a 171-band reflectance signature. Raw digital numbers were converted to percent reflectance using standard white/dark calibration with a 99% Spectralon panel as the white reference. The dark reference was acquired by completely blocking the lens aperture with an opaque lens cap, thereby recording the detector’s thermal and electronic background signal under the same acquisition settings. A human expert then delineated regions of interest (ROIs) in Environment for Visualizing Imagery (ENVI) software version 4.8. to establish the pixel-level ground truth, resulting in 52 calibrated images and 295,340 labeled target pixels.

The task is pixel-wise FPO classification, in which each target pixel is assigned to one of C=13 classes: *fillet* tissue and 12 FPO material classes commonly encountered in poultry processing (ABS, FAB, HDPE, LDPE, NYL, PET, PP, PS, PUR, PVC, RUB, and TEF). To represent within-class variability arising from different source materials, six different material sources were included for each of PET, PP, PS, PVC, LDPE, and HDPE; three sources were included for each of PUR, RUB, FAB, NYL, and TEF; one source was used for ABS [37]. In addition to the material-source variability described above, the FPO samples exhibited differences in visual appearance within individual material classes. For example, representative PET samples included white, dark-blue, and pink pieces [37]. Such visible differences do not necessarily correspond directly to differences in material class. Within the 1000–1700 nm NIR range considered in this study, the measured spectral response is primarily influenced by wavelength-dependent absorption and scattering associated with the material composition and structure, while pigmentation, additives, material thickness, and surface characteristics can further affect the observed reflectance magnitude. Consequently, samples originating from different sources may exhibit variations in both visible appearance and reflected intensity while retaining material-specific spectral characteristics that support discrimination from poultry tissue and among FPO classes. The source materials were cut into standardized pieces of approximately 5×5 mm and placed randomly but uniformly over the exposed surfaces of fresh skinless, boneless broiler breast fillets (n=52), providing coverage across different tissue regions, including muscle and visible fat deposits. Background and conveyor pixels were excluded through ROI selection so that the analysis focused specifically on tissue-versus-FPO discrimination and FPO-type classification. The data partitioning is performed at the labeled target-pixel level rather than at the hyperspectral-image level, following the commonly used pixel-level evaluation protocol in HSI classification studies with a limited number of hyperspectral images. Each hyperspectral image contains fillet tissue together with pieces from a single FPO class originating from one material source. For FPO classes represented by multiple source materials, separate hyperspectral images were acquired using pieces from different sources. Accordingly, training and validation samples were drawn across the available hyperspectral images to ensure representation of all 13 classes and the available within-class source variability. Class-balanced random sampling was used to select 2400 labeled target pixels per class for training (31,200 total) and 1200 pixels per class for validation (15,600 total), while all remaining labeled target pixels were assigned to the test set (248,540 total). During patch extraction, each labeled target pixel defines the center of a 7×7×171 spatial–spectral cube. Consequently, individual hyperspectral images may contribute labeled target pixels to multiple data partitions. All metrics are reported over ten independent random seeds and are presented as the mean ± standard deviation.

### 3.2. Model Architecture

**Figure 1:**
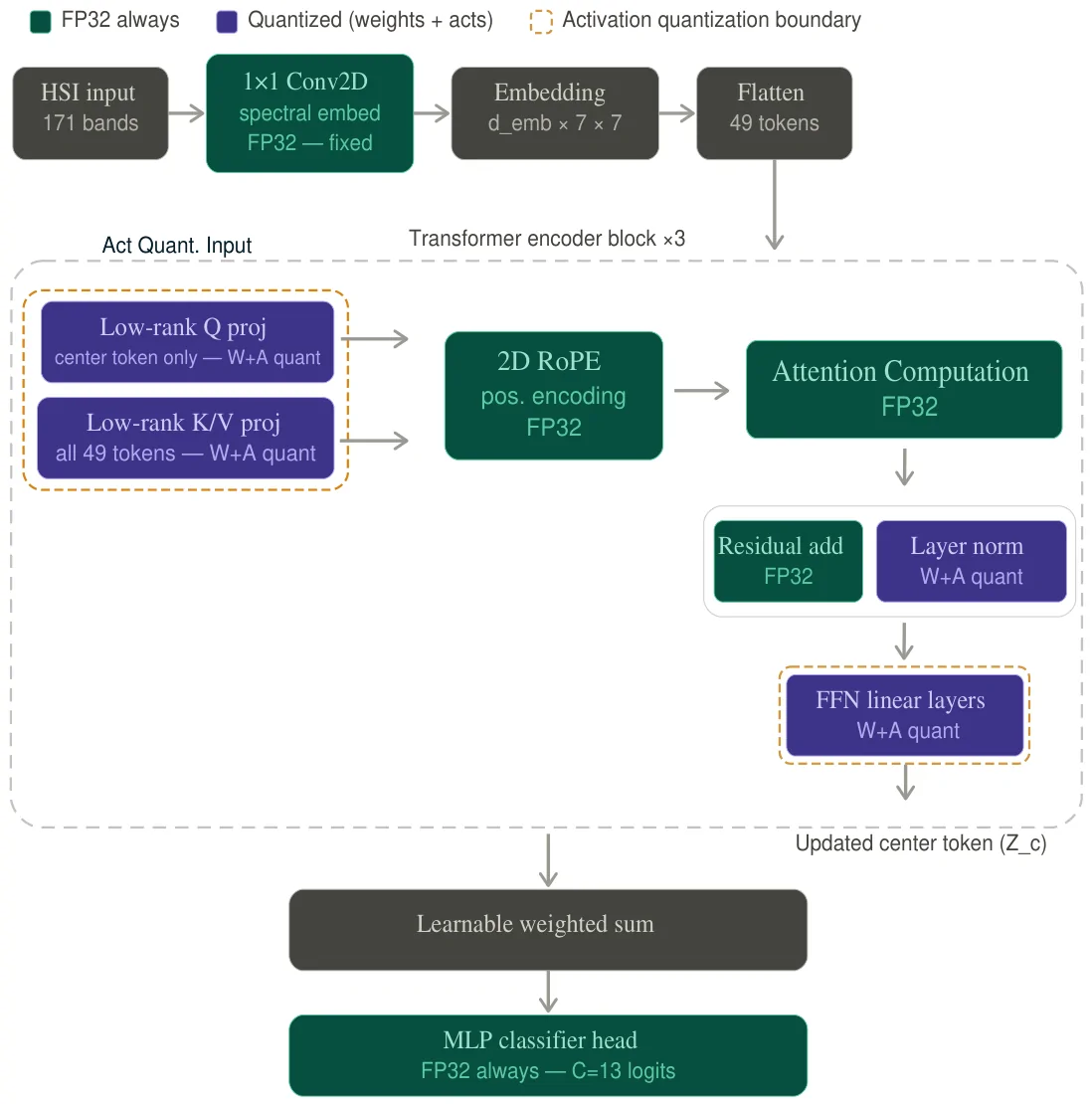
Proposed mixed-precision quantization strategy and computation flow of the FPO classification spatial–spectral transformer architecture. FP32-retained operations and quantized weight/activation modules are distinguished by color, while dashed boundaries indicate activation–quantization transitions. The forward path illustrates how activations move between FP32 and target low-precision regions across the spectral embedding, transformer encoder blocks, attention operations, and classifier head. During FQT, the same module-level precision allocation is maintained during backpropagation, with designated quantized modules operating in the target precision and selected numerically sensitive or implementation-constrained modules retained in FP32.

We use a state-of-the-art transformer-based spatial–spectral framework as the fixed backbone for pixel-level FPO classification on poultry in all quantization experiments (Figure 1; see [37]). For each labeled target pixel, a 7×7 neighborhood patch is extracted, and each pixel within the patch is represented by its 171-band reflectance vector. A pixel-wise 1×1 convolution, equivalently a linear projection applied independently at each spatial location, maps the 171-dimensional spectrum to a $d_{\mathrm{emb}}$-dimensional embedding, thereby producing a tensor in $R^{d_{\mathrm{emb}}\times 7\times 7}$.

Each resulting 7×7 embedding grid is flattened into a sequence of n=49 tokens and passed through three transformer encoder blocks. Each block incorporates three key components: (i) low-rank factorization of the Q/K/V and output projection matrices to reduce parameter count, (ii) center-focused linear attention (CFLA), in which only the center token serves as the query while all *n* tokens provide keys and values, thereby reducing the attention complexity from quadratic to linear in *n*, and (iii) patch-local mixed-axis 2D RoPE with learnable frequencies to encode relative spatial offsets within the local patch. After each block, the updated center-token embedding replaces the previous center token before being passed to the next block. In addition, the center-token outputs from all three blocks are retained and fused through a learnable weighted sum. The resulting fused representation is then passed to a two-layer MLP head to produce logits over the C=13 classes, corresponding to 12 polymer types and fillet tissue (see [37] for details).

### 3.3. Quantization Settings and Mixed-Precision Strategy

Building on the general quantization framework described in Section 2.2, this section specifies how FQT and PTQ are instantiated within the proposed transformer architecture, including the module-level mixed-precision allocation and precision-specific execution settings used throughout the experiments. Across all experiments, the model architecture, optimization hyperparameters, and precision-allocation strategy are kept strictly fixed; only the numerical formats and their corresponding scaling mechanisms are varied, as shown in Figure 1. The mixed-precision module allocation was defined based on architectural role, computational characteristics, numerical sensitivity, and implementation compatibility. Quantization is applied to the linear projection layers and layer-normalization modules within the transformer encoder, thereby targeting the core representation-learning path of the model. The initial 1×1 Conv2D feature extractor is retained in FP32 because it operates directly on the hyperspectral input and lies outside the transformer backbone, allowing the quantization effects to remain focused on the transformer encoder. RoPE and Softmax are also retained in FP32 because they are non-GEMM operations with limited opportunity for low-precision hardware acceleration, while their outputs directly affect positional encoding and normalized attention weights. Similarly, the final MLP classifier head is retained in FP32 because it is a task-specific output module outside the core transformer encoder. In addition, its final projection produces C=13 class logits, which introduces alignment constraints for the low-precision Transformer Engine implementation used in this study without additional padding or architectural modification. Thus, the adopted strategy applies reduced precision primarily within the transformer representation-learning stages while preserving selected input, attention, and output operations in FP32. The overall effect of this module selection is further examined through the NVFP4 ablation in Section 4.5. Figure 1 summarizes the resulting precision flow, with activations transitioning between the designated low-precision and FP32 regions according to the module-allocation strategy described above. During FQT, this same precision allocation is maintained during both the forward and backward passes. Accordingly, throughout this study, mixed-precision FQT denotes native target-precision training of the selected quantized modules, while the designated FP32 modules remain in full precision according to the module-allocation strategy described above.

- **FQT (training + inference):** Under the FQT paradigm, the model is trained end-to-end with the target numerical format active during both the forward and backward passes. We evaluate five precision modes: FP32 (baseline), FP16, BF16, FP8, and FP4. For the 16-bit unscaled formats, FP16 and BF16 arithmetic are applied dynamically using simple casting to target precision. To mitigate gradient underflow in FP16 due to two fewer *E* bits compared to FP32, dynamic loss scaling is employed during backpropagation. For the ultra-low-precision modes FP8 and FP4, weights, activations, and gradients involved in GEMMs are quantized dynamically during execution. FP8 uses the per-tensor current-scaling formulation in Equation (3), switching dynamically between the E4M3 format for the forward pass and the higher-dynamic-range E5M2 format for gradient computation. FP4 uses the E2M1 format governed by the two-level block scaling mechanism in Equation (4). In addition, FQT records training-time statistics and metadata (e.g., $X_{\min},X_{\max}$ or, equivalently, max(|X|)), allowing inference to reuse these statistics to determine the scaling factor σ without the need for a separate calibration dataset.
- **PTQ (inference only):** The PTQ paradigm confines quantization exclusively to the inference stage, thereby simulating practical deployment conditions. All PTQ experiments are initialized from a seed-matched pretrained FP32 baseline checkpoint, which reflects the typical real-world PTQ workflow. The model then undergoes a calibration phase consisting of a forward-only pass over a calibration dataset. In our experiments, the calibration set comprises 5% of the data split designated for calibration. This step estimates the activation ranges required to derive the scaling factors σ for the respective target formats. For the 16-bit formats, weights and activations are statically cast to the target precision. For FP8 and FP4, by contrast, weights and activations are dynamically quantized at runtime using the calibration statistics before evaluation on the held-out test set.

### 3.4. Training and Inference Protocols

To ensure a rigorous and fair comparison across all precision modes, an identical training configuration is enforced throughout. Models are optimized using Adam with a fixed learning rate of $\eta =10^{-3}$ and zero weight decay (λ=0). No learning-rate scheduler is used. Training proceeds for a maximum of 200 epochs, with early stopping triggered when validation overall accuracy fails to improve for 150 consecutive epochs. The batch size is fixed at 512 for all modes. Input hyperspectral data are normalized using a global min–max scaler derived exclusively from the training split and then applied uniformly to the validation and test splits. No data augmentation is used. Importantly, all hyperparameters are tuned only on the FP32 baseline, and no precision-specific tuning is performed, ensuring that any observed performance differences are attributable to quantization behavior rather than separately optimized settings. To ensure reproducibility, all computational libraries and environments are seeded before data loading and model initialization, and deterministic backend algorithms are enforced. Consistent with the dataset evaluation protocol, each precision mode is evaluated over 10 independent predefined random seeds, and all reported results are presented as the mean ± standard deviation across these runs. Similarly, to ensure a rigorous and fair comparison between FQT and PTQ at inference time, an identical inference configuration is enforced. Both inference flows use the same mixed-precision assignment of layers and operators, as well as the same target low-precision format for the quantized components. All quantized models are evaluated on the same test set using identical batch sizes. In addition, for accurate efficiency measurement, each benchmark includes 10 warm-up passes before timing.

### 3.5. Evaluation Metrics

To comprehensively assess the impact of reduced-precision floating-point formats on the hyperspectral FPO classification task, we evaluate the models along four complementary dimensions: **model size reduction**, **predictive accuracy**, **practical training efficiency**, and **practical inference efficiency**. Model size reduction is reported for both FQT and PTQ. However, for a given target precision, the resulting quantized model footprint is identical across the two paradigms because both employ the same mixed-precision allocation strategy and the same target numerical formats for quantized components. Predictive accuracy is evaluated for both FQT and PTQ, training efficiency is evaluated for FQT only, and inference efficiency is evaluated for both FQT and PTQ. Together, these measurements enable a unified assessment of accuracy–efficiency trade-offs under different precision regimes.

#### 3.5.1. Model Size Reduction

For each target precision, we report the on-disk storage footprint (in MB) of the serialized quantized model weights as a measure of compression. Because FQT and PTQ use the same mixed-precision strategy and quantize the same subset of layers to the same target format, the resulting stored model size is the same for both paradigms at a given precision level. Therefore, model size reduction is reported once per target precision rather than separately for FQT and PTQ. This metric provides a practical view of deployment-time compression benefits independent of whether the low-precision model was obtained through training-time quantization or post-training conversion.

#### 3.5.2. Accuracy Metrics

Classification performance is evaluated on the held-out test set using standard multi-class metrics derived from the confusion matrix. Let *C* denote the K×K confusion matrix, where $C_{ij}$ represents the number of samples whose true class is *i* and predicted class is *j*, and let *N* denote the total number of test samples. We report three primary scalar metrics:

- **Overall Accuracy (OA):** The proportion of correctly classified samples over all classes:
  (5) $$\mathrm{OA}=\frac{1}{N}\sum_{k=1}^{K}C_{kk}.$$
- **Average Accuracy (AA):** The unweighted mean of per-class recall, ensuring that all classes contribute equally regardless of class frequency:
  (6) $$\mathrm{AA}=\frac{1}{K}\sum_{k=1}^{K}\frac{C_{kk}}{\sum_{j=1}^{K}C_{kj}}.$$
- **Cohen’s Kappa (κ):** A chance-corrected agreement measure defined as
  (7) $$\kappa =\frac{p_{o}-p_{e}}{1-p_{e}},$$
  where $p_{o}$ is the observed agreement, equivalent to OA, and $p_{e}$ is the agreement expected by chance.

All accuracy metrics are reported as percentages. In addition, per-class recall is also recorded to analyze class-specific degradation patterns induced by quantization noise.

#### 3.5.3. Training-Time Efficiency Metrics (FQT Only)

For models trained under the mixed-precision FQT paradigm, we measure a set of hardware- and optimization-oriented metrics to quantify the practical computational effects of reduced-precision training. In addition, to examine the trade-off between predictive performance and training efficiency under different target precisions, we evaluate training behavior across a batch-size sweep from $2^{6}$ to $2^{14}$. These metrics are monitored throughout the training process:

- **Peak GPU Memory:** The maximum allocated accelerator memory (in MB) observed during training, reflecting the largest memory footprint required for weights, activations, gradients, and optimizer states. Peak-memory statistics are reset at the beginning of each epoch.
- **Total Training Time:** The total end-to-end wall-clock training time.
- **Training Throughput:** The number of samples (7×7 hyperspectral cubes) processed per second during training, computed for each epoch and then averaged across epochs.
- **Energy Consumption:** The total estimated GPU energy usage (in Wh), obtained by periodically sampling hardware power draw and integrating the resulting power trace over the full training duration.
- **Convergence Speed:** The number of epochs required for validation OA to reach 90%, 95%, and 99% of the model’s peak validation OA, providing a proxy for how reduced precision affects the optimization trajectory.
- **Loss Stability:** The smoothness of optimization, quantified as the rolling standard deviation of the training loss over the entire training loop.

#### 3.5.4. Inference-Time Efficiency Metrics (FQT and PTQ)

To assess deployment viability, inference-time efficiency is evaluated for all precision modes under both FQT and PTQ. Measurements are collected over a standardized benchmarking window of 1000 consecutive forward passes, preceded by 30 warm-up passes to ensure stable hardware conditions. To characterize inference behavior under different workloads, we additionally evaluate performance across a batch-size sweep from $2^{0}$ to $2^{14}$. The following metrics are reported:

- **Latency:** The mean wall-clock time required to complete a single forward pass at a given batch size, reported in milliseconds (ms).
- **Inference Throughput:** The number of samples processed per second at each evaluated batch size.
- **Peak Inference Throughput:** The maximum throughput achieved across all tested batch sizes, indicating the highest effective hardware utilization for a given precision mode.
- **Peak Inference Memory:** The maximum accelerator memory allocated strictly during forward-pass execution.
- **Inference Energy Efficiency:** The total GPU energy consumed during the timed benchmarking window, normalized by the number of processed samples and reported as energy per sample (Wh/sample).

### 3.6. Hardware and Software Environment

All experiments were conducted on a single NVIDIA GeForce RTX 5080 Laptop GPU (Santa Clara, CA, USA) based on the Blackwell SM 12.0 architecture, with 16,302.6 MiB of GDDR7 memory. The device provides tensor cores with native support for FP8 and FP4 arithmetic. The system used NVIDIA driver 591.74 and CUDA 12.9. The software environment consisted of Python 3.12.12, PyTorch 2.10.0+cu129, cuDNN 9.1.0.2, and NVIDIA Transformer Engine 2.11.0. All experiments were executed under WSL2 with Linux kernel 6.6.87.2.

## 4. Results and Analysis

This section first examines model compression under the common mixed-precision design to quantify how checkpoint size changes as the target numerical precision is reduced. We then evaluate predictive performance under mixed-precision fully quantized training to determine how far precision can be lowered in the compact transformer-based classifier without materially degrading hyperspectral foreign plastic object classification. Next, we compare fully quantized training and post-training quantization at matched target precisions in terms of classification accuracy. We then analyze the training dynamics and convergence stability of fully quantized training, followed by its training-time efficiency and batch-size scaling behavior on the Blackwell GPU platform. Finally, we compare the inference-time behavior of fully quantized training and post-training quantization on the same hardware. Unless otherwise stated, the main results correspond to the default configuration used for the headline experiments, whereas the scaling studies isolate the effect of batch size.

### 4.1. Model Compression and Serialized Footprint

Table 1 reports the on-disk serialized model size obtained at each target precision under the common mixed-precision strategy. Because FQT and PTQ apply the same precision allocation to the same subset of layers, the resulting stored model size is identical for both paradigms at a given target precision. Accordingly, model size is reported once per precision level as a deployment-oriented compression metric rather than separately for FQT and PTQ.

A clear monotonic reduction in model size is observed as the precision of the quantized components decreases. Relative to the FP32 reference checkpoint (1.36 MB), FP16 and BF16 reduce the model size to 0.79 MB, corresponding to a compression ratio of 1.71×. FP8 further reduces the checkpoint size to 0.51 MB (2.67× compression), whereas NVFP4 produces the smallest artifact at 0.37 MB, corresponding to 3.69× compression. The identical sizes of FP16 and BF16 are expected because both use 16 bits for the quantized portion under the same mixed-precision assignment. Similarly, the progressive reduction from FP16/BF16 to FP8 and then to NVFP4 directly reflects the lower storage cost of the quantized weights in the transformer backbone. The table further shows that scale metadata contributes negligibly to the total model size. For FP8 and NVFP4, the scale overhead is less than 0.01 MB in both cases, indicating that the storage gains from lower-precision quantization are not meaningfully offset by the additional quantization parameters. At the same time, the retained FP32 portion remains fixed at 0.23 MB across all quantized variants, which establishes a lower bound on the total achievable model size under the adopted selective mixed-precision design. This behavior is consistent with the quantization strategy described in Section 3.3, in which numerically sensitive components are preserved in FP32 while only the dominant transformer operations are quantized.

### 4.2. Accuracy Analysis of FQT and PTQ

#### 4.2.1. Predictive Performance Under Mixed-Precision FQT

We first focus on the FQT rows in Table 2 to isolate the effect of reduced precision during training. Overall, the results show that the proposed mixed-precision design remains robust across the full tested precision spectrum for the compact transformer-based classifier on this hyperspectral FPO classification task. Relative to the FP32 baseline (98.88% OA), all reduced-precision FQT configurations remain within 0.63 percentage points (pp), indicating that most of the predictive performance can be preserved even when the dominant GEMM-intensive components of the transformer are executed in lower precision.

Among the reduced-precision FQT modes, BF16 provides the closest match to FP32, achieving 98.60% OA, 98.98% AA, and 98.45% κ, corresponding to only a 0.28 pp reduction in OA. FP8 is the next strongest low-precision setting, with 98.53% OA, 98.97% AA, and 98.37% κ, representing a 0.35 pp drop relative to FP32. FP16 follows with 98.33% OA, corresponding to a 0.55 pp reduction, whereas NVFP4 yields the lowest accuracy among the FQT variants at 98.25% OA, a 0.63 pp decrease from the baseline. Importantly, however, even the most aggressive tested format does not produce a severe loss in predictive performance. The same overall ordering is preserved across AA and κ, indicating that the observed trend is consistent not only in aggregate accuracy but also in class-balanced performance and chance-corrected agreement.

**Figure 2:**
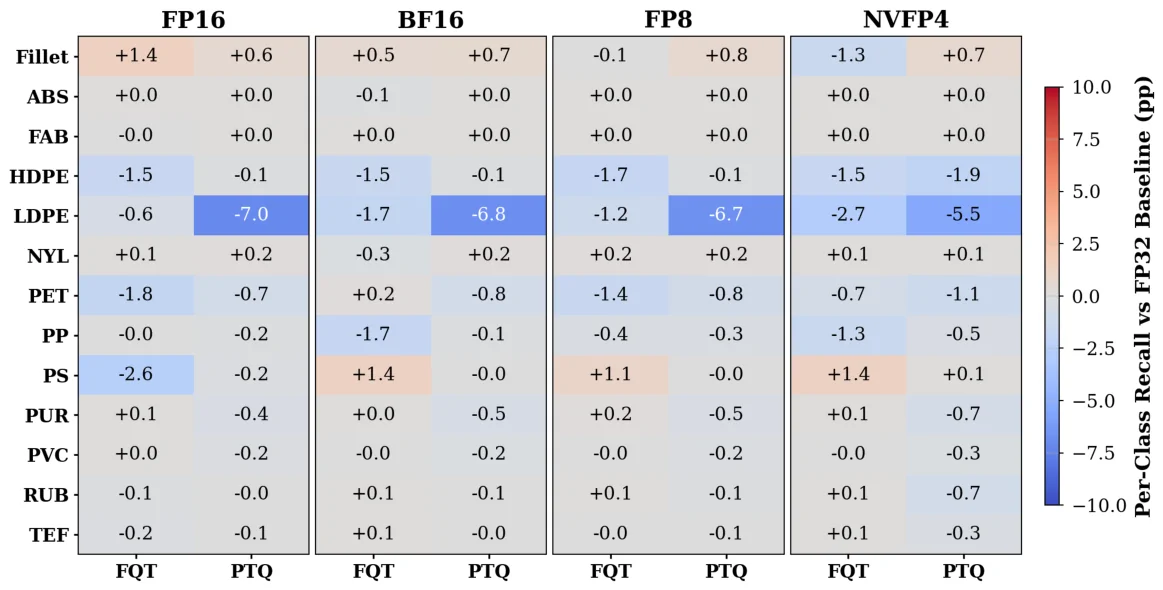
Per–class recall change relative to the FP32 baseline. Each panel corresponds to a target precision; within each panel, FQT and PTQ columns reveal which classes benefit from training-time adaptation versus post-training calibration.

The class-wise results in Figure 2 further clarify how quantization affects predictive behavior under FQT. The FQT columns show that the impact of reduced precision is not uniform across classes, but instead is concentrated in a subset of materials. In particular, larger recall reductions are observed for classes such as HDPE, LDPE, and PET, whereas several other classes remain nearly unchanged across all tested precisions. At the same time, a few classes exhibit slight recall improvements relative to the FP32 baseline, most notably PS under BF16, FP8, and NVFP4, and fillet under FP16 and BF16. This heterogeneous pattern is consistent with the nature of hyperspectral classification, where discriminative information may be encoded in subtle reflectance differences across correlated spectral bands, making some classes more sensitive than others to quantization noise.

Several conclusions follow from these results. First, the transformer-based hyperspectral classifier is reasonably tolerant to low-precision training when numerically sensitive components are preserved in FP32 and quantization is applied selectively to the dominant transformer operations. Second, aggressive quantization does not lead to catastrophic failure on this task, even for NVFP4, although the ranking of formats suggests that preserving dynamic range and effective scaling remains important for maintaining fine-grained spatial–spectral discrimination. Third, the fact that FP8 slightly outperforms FP16 in this setting is noteworthy. A plausible explanation is that the tensor-level scaling used for FP8 may better adapt numerical ranges to the underlying activation distributions than FP16, which, in our setup, primarily relies on conventional mixed-precision execution with loss scaling. Although this interpretation is consistent with the observed behavior, it should be viewed as a task- and implementation-specific explanation rather than a universal ordering between FP16 and FP8.

#### 4.2.2. Comparative Accuracy Analysis of FQT and PTQ

Having established the feasibility of low-precision FQT, we next compare FQT and PTQ at matched target precisions using Table 2. A clear and consistent pattern emerges: FQT outperforms PTQ at every tested reduced precision. At BF16, FQT improves OA over PTQ by 0.52 pp (98.60% versus 98.08%). The corresponding OA gains are 0.31 pp for FP16 (98.33% versus 98.02%), 0.44 pp for FP8 (98.53% versus 98.09%), and 0.33 pp for NVFP4 (98.25% versus 97.91%). The same ordering is preserved for AA and κ, indicating that the advantage of FQT is consistent across overall accuracy, class-balanced performance, and chance-corrected agreement.

The class-wise recall differences in Figure 2 provide additional insight into how the two quantization flows differ. Across the tested precisions, the FQT columns show that deviations from the FP32 baseline are distributed across a small subset of classes, most notably HDPE, LDPE, PET, and, in some cases, PS. By contrast, the PTQ columns indicate that the degradation is more concentrated, with LDPE showing the most pronounced and persistent recall drop across precisions. This class-specific pattern suggests that training-time exposure to quantization noise enables FQT to distribute the impact of reduced precision more smoothly across the learned representation, whereas PTQ is more vulnerable to sharper degradation in particularly sensitive classes.

Although both flows remain highly accurate in absolute terms, the gap between FQT and PTQ is practically meaningful. All PTQ variants remain within 0.97 pp OA of the FP32 baseline, confirming that PTQ is still a strong and viable low-overhead deployment strategy for this task. However, the uniformly better results of FQT indicate that calibration alone is less effective than training-time adaptation for absorbing low-precision noise in this hyperspectral setting. This observation is especially relevant for compact domain-specific models trained from scratch on high-dimensional spectral data, where the learned representation may be less redundant and therefore more sensitive to quantization perturbations than that of very large pretrained models.

From a deployment perspective, the matched-precision comparison supports a clear interpretation. When retraining is feasible and preserving accuracy is important, FQT is preferable to PTQ, particularly at more aggressive precisions such as FP8 and NVFP4. When retraining is impractical, PTQ remains a practical fallback because it preserves strong absolute accuracy while requiring only a calibration stage. Nonetheless, this convenience comes with a consistent accuracy penalty relative to FQT and a stronger dependence on the quality and representativeness of the calibration data.

#### 4.2.3. Convergence Behavior and Training Stability Under FQT

**Figure 3:**
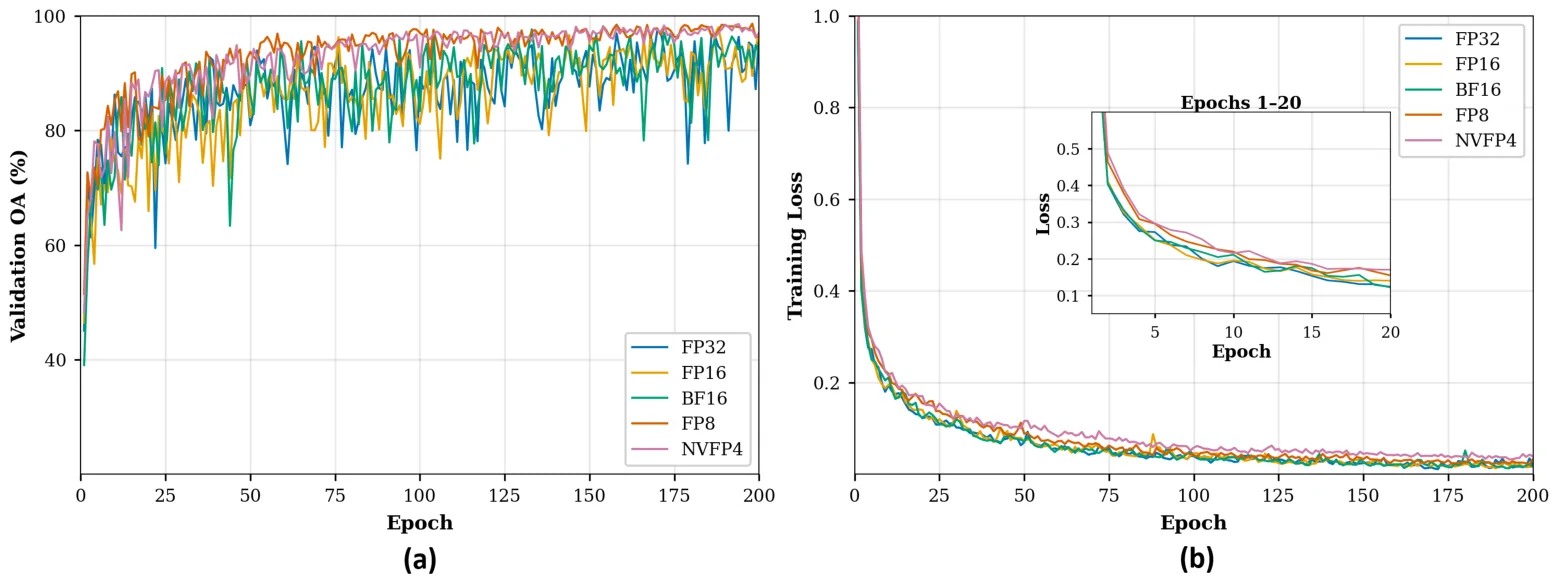
FQT convergence trajectories. (**a**) Validation OA vs. epoch, showing validation OA convergence. (**b**) Training loss vs. epoch, showing training loss stability; the inset highlights early-phase behavior in low-precision modes.

The convergence statistics in Table 3, together with the trajectories in Figure 3a, show that reduced precision does not destabilize optimization under the mixed-precision formulation. On the contrary, all reduced-precision modes reach 90% of their own peak validation OA in fewer epochs than FP32. FP8 converges fastest to both 90% and 95% of its own peak validation OA, requiring only 12 and 33 epochs, respectively, compared with 20 and 43 epochs for FP32. BF16 shows similarly rapid convergence, reaching the same thresholds in 14 and 34 epochs. At the stricter 99% threshold, FP16 converges fastest at 75 epochs, followed by FP8 at 87 and NVFP4 at 96, all substantially ahead of FP32 at 141 epochs.

These convergence results should be interpreted carefully. Because the thresholds are defined relative to each format’s *own* peak validation OA, faster convergence does not imply that lower precision reaches a better optimum than FP32. Rather, it indicates that the reduced-precision models attain a high fraction of their eventual validation performance in fewer training epochs. In this task, such behavior suggests that low-precision training can learn useful spatial–spectral discriminative structure efficiently, even if the final optimum remains slightly below that of the full-precision baseline. One plausible interpretation is that quantization noise acts as a mild implicit regularizer, accelerating coarse optimization while slightly constraining the final attainable optimum at more aggressive precisions.

Figure 3b provides the complementary loss-level view and further supports the numerical stability of the training process. All precision modes exhibit a consistent downward loss trajectory without major divergence or irregular behavior. The inset over epochs 1–20 shows that FP8 and NVFP4 have somewhat higher and slightly noisier early-stage losses than FP32, FP16, and BF16, but this additional variability does not prevent stable optimization or high final accuracy. By later epochs, all modes settle into similar low-loss regimes, although NVFP4 remains modestly higher than the others, which is consistent with its slightly lower final test accuracy. Taken together, these results indicate that the given mixed-precision training strategy remains numerically stable across all evaluated floating-point precisions while enabling convergence rates that are competitive with, and in some cases faster than, FP32.

### 4.3. Training-Time Efficiency Under FQT

#### 4.3.1. Training-Time Efficiency at the Default Batch Size

Table 4 summarizes training-time efficiency under FQT at the default batch size of 512. The results show that reduced precision yields clear memory savings, but these savings do not translate uniformly into higher end-to-end training throughput for this compact hyperspectral transformer. Relative to FP32, FP16 and BF16 reduce peak memory from 306.6 MB to 272.3 MB, corresponding to a 1.13× reduction, while maintaining essentially the same throughput: 23,397 and 23,618 samples/s, respectively, versus 23,320 samples/s for FP32. Their total training time remains unchanged at 0.09 h, and both slightly reduce total energy consumption, with FP16 yielding the lowest energy usage among the 16/32-bit modes at 2.18 Wh.

FP8 and NVFP4 provide larger memory reductions, lowering peak memory to 247.1 MB and 237.3 MB, respectively, equivalent to 1.24× and 1.29× reductions relative to FP32. However, these gains are accompanied by substantially lower throughput at the default workload. FP8 reaches 14,804 samples/s, corresponding to a 0.63× speedup relative to FP32, while NVFP4 reaches 12,779 samples/s, or 0.55× relative throughput. Their longer training times, 0.15 h for FP8 and 0.16 h for NVFP4, also increase total energy consumption to 3.03 Wh and 2.86 Wh, respectively. Thus, at the default batch size, ultra-low precision improves memory efficiency but does not improve training throughput or energy efficiency.

This behavior is consistent with the workload characteristics of the model and the adopted mixed-precision design. Because only selected modules are quantized and the network itself is relatively compact, the arithmetic intensity of the low-precision GEMMs is likely insufficient to amortize the additional overhead associated with dynamic scaling, quantization, dequantization, and format conversion. In other words, the memory benefit of FP8 and NVFP4 appears immediately, whereas the throughput benefit requires a larger effective workload so that the low-precision tensor-core kernels can dominate execution more fully. This differs from the regime encountered in very large transformer models, where GEMMs are sufficiently large for low-precision hardware acceleration to provide more consistent end-to-end gains.

#### 4.3.2. Training Batch-Size Scaling

**Figure 4:**
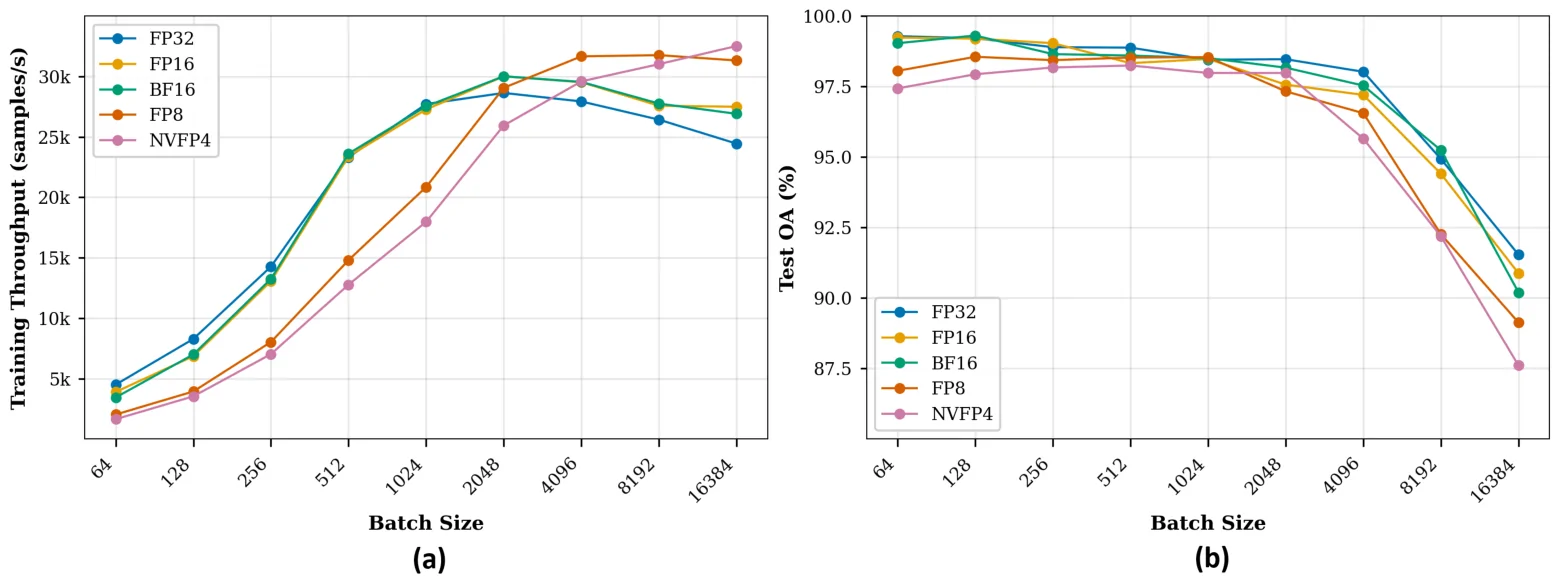
Training scaling behavior across batch sizes [64, 128, 256, 512, 1024, 2048, 4096, 8192, 16384]. (**a**) Training throughput (samples/s) vs batch size; (**b**) test OA (%) vs batch size.

The batch-size sweep in Figure 4 clarifies when low-precision hardware support begins to provide a practical throughput advantage during training. As shown in Figure 4a, FP32, FP16, and BF16 remain competitive or superior at small and moderate batch sizes. Up to batch size 2048, the best throughput is still achieved by FP16 or BF16, with BF16 reaching 30,017 samples/s at batch size 2048, while FP8 remains slightly lower at 29,061 samples/s and NVFP4 at 25,949 samples/s. At batch size 512, which is the default setting used in the main experiments, BF16 again provides the highest throughput, followed closely by FP16 and FP32, whereas FP8 and NVFP4 remain substantially slower.

A clear crossover emerges only at larger batch sizes. At batch size 4096, FP8 becomes the fastest configuration at 31,664 samples/s, exceeding FP32 at 27,931 samples/s and also surpassing FP16 and BF16. This advantage persists at batch size 8192, where FP8 again leads at 31,764 samples/s. At the largest tested batch size of 16,384, NVFP4 becomes the fastest configuration at 32,520 samples/s, slightly ahead of FP8 at 31,321 samples/s. These results indicate that the throughput advantage of native FP8/FP4 tensor-core execution is strongly workload-dependent and becomes visible only once the GEMM dimensions are large enough for the specialized hardware to offset the overhead of low-precision scaling and runtime quantization.

The key question, however, is whether these throughput gains during training are obtained without compromising predictive performance. Figure 4b shows that they are not. As batch size increases beyond the moderate regime, test OA declines for all precisions, with the degradation becoming more pronounced for the more aggressive low-precision formats. At batch size 4096, FP32 still retains 98.02% OA, whereas FP8 falls to 96.55% and NVFP4 to 95.65%. At batch size 8192, all precisions degrade further, and by batch size 16,384, the drop becomes substantial across the board, with NVFP4 reaching 87.61% OA. Thus, the throughput advantage of FP8 and NVFP4 emerges primarily in a regime where optimization quality has already begun to deteriorate, especially for the lower-precision modes.

Taken together, these results suggest that the most practically useful operating region for this model or any small model is not the absolute maximum-throughput point, but rather the intermediate batch-size regime that balances computational efficiency and predictive accuracy. More broadly, the findings reinforce an important systems-level conclusion: native FP8 and FP4 hardware support does not guarantee end-to-end training acceleration for compact, domain-specific transformers. Instead, the benefit appears only after sufficient workload scaling, and even then it must be weighed against the accompanying reduction in predictive performance.

### 4.4. Inference-Time Efficiency Under FQT and PTQ

#### 4.4.1. Inference Batch-Size Scaling

We next examine inference-time efficiency for the quantized models obtained from the two quantization flows. Because FQT and PTQ use the same mixed-precision assignment, quantize the same subset of operations, and target the same numerical formats, their inference behavior should in principle be very similar at a matched precision. Accordingly, the main factors governing inference efficiency are not when the model is quantized, but rather the target numerical format and, most importantly, the batch size used at deployment.

**Figure 5:**
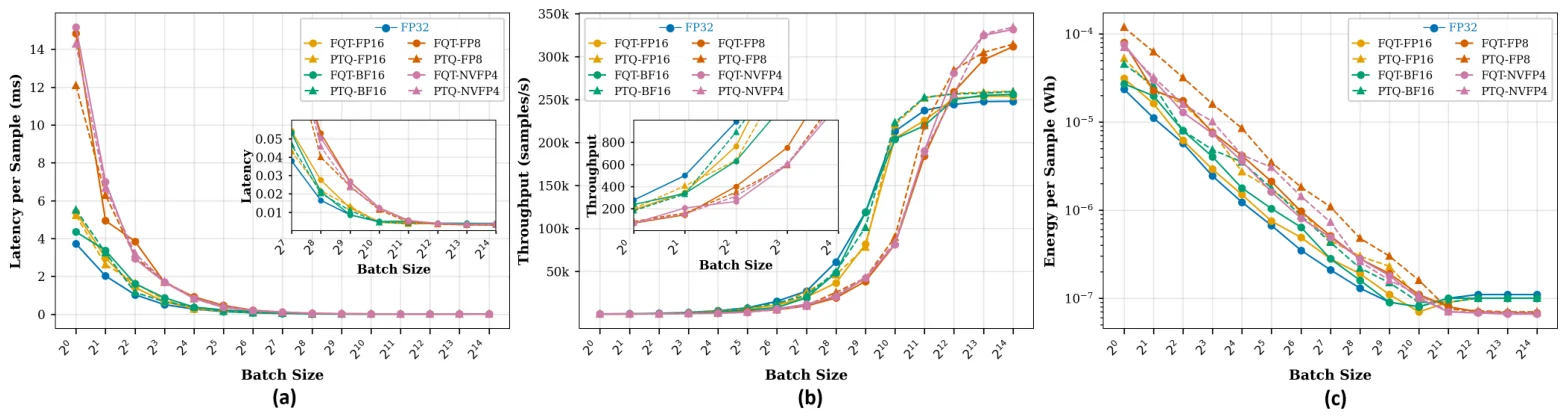
Inference scaling across batch sizes $2^{0}$–$2^{14}$ for FQT (solid lines) and PTQ (dashed lines), using the model trained at batch size b=512. (**a**) Latency per sample vs. batch size (ms/sample). (**b**) Throughput vs. batch size (samples/s). (**c**) Energy per sample vs. batch size (Wh, $\log_{10}$ scale). Each color represents one target precision mode. Solid lines: FQT; dashed lines: PTQ.

Figure 5 shows that batch size is the dominant driver of inference efficiency across all precisions and both flows. In Figure 5a, latency per sample decreases sharply as batch size increases, especially from $2^{0}$ to approximately $2^{8}$–$2^{10}$, after which the gains begin to taper. Figure 5b shows the complementary throughput view: at small batch sizes, FP32 and the 16-bit modes remain competitive or superior, whereas the lower-precision FP8 and NVFP4 modes lag behind. This behavior mirrors the training-time results at small workloads and indicates that, for compact models, the overhead associated with scaling, format conversion, and runtime quantization can dominate when the effective GEMM workload is too small to fully utilize the low-precision tensor-core path.

At larger batch sizes, however, the trend reverses. As the batch size increases, FP8 and especially NVFP4 achieve higher throughput than FP32 and the 16-bit modes in both FQT and PTQ. This crossover is consistent with the increasing arithmetic intensity of batched inference: once the workload becomes sufficiently large, the specialized low-precision hardware is better able to amortize its overhead and deliver higher effective throughput. Importantly, unlike the training setting, increasing the inference batch size does not introduce an optimization-related accuracy penalty, because the model parameters are fixed and no learning occurs during deployment. Consequently, the throughput gains from larger inference batches can be harvested without the accuracy degradation that accompanied large-batch training, although they still come with the usual system-level trade-off of higher batch latency and reduced responsiveness for individual requests.

The energy-per-sample curves in Figure 5c reinforce the same conclusion. Across all flows and precisions, energy per sample decreases by orders of magnitude as batch size increases, indicating that batching improves not only throughput but also energy efficiency. By the largest batch sizes, the curves begin to converge, suggesting that batching has a stronger effect on deployment energy efficiency than precision alone. In this regime, lower-precision formats still provide a modest additional advantage, but their main runtime benefit appears only once batch sizes are large enough for the workload to become hardware-efficient.

#### 4.4.2. Peak Throughput and Deployment Implications

Table 5 summarizes the peak inference throughput achieved by each configuration. Two observations stand out. First, the differences between FQT- and PTQ-derived models at the same precision are very small, which is consistent with the expectation that their inference behavior should be nearly identical under a shared mixed-precision deployment policy. For example, peak throughput differs only marginally between FQT–FP8 and PTQ–FP8 (311,555 vs. 314,524 samples/s) and between FQT–NVFP4 and PTQ–NVFP4 (331,545 vs. 334,165 samples/s). These small gaps are better interpreted as implementation- and measurement-level variation than as a fundamental difference between the two quantization flows at inference time.

Second, the highest peak throughput is achieved not by the 16-bit modes, but by the more aggressive low-precision formats at the largest tested batch size. Relative to the FP32 reference peak of 247,999.6 samples/s, FQT–FP16 and FQT–BF16 provide only modest gains of 1.02× and 1.03×, while PTQ–FP16 and PTQ–BF16 reach 1.05×. By contrast, FP8 reaches approximately 1.26×–1.27× the FP32 peak, and NVFP4 attains the highest overall throughput, achieving 1.34×–1.35× speedup over FP32. Notably, all configurations reach their peak throughput at the largest tested batch size, $b^{*}=16384$, further emphasizing that the hardware advantage of FP8 and NVFP4 emerges only in the high-workload regime.

Taken together, these results suggest a more nuanced inference story than the training results. At small deployment batch sizes, lower precision does not automatically yield better inference performance, because overheads dominate for this compact transformer-based hyperspectral model. At large batch sizes, however, FP8 and especially NVFP4 do provide the highest throughput and lowest energy per sample. Since inference batching does not alter model accuracy in the way that large-batch training can, these gains are practically attainable whenever the deployment scenario can tolerate large batched execution. Therefore, if the primary objective is maximum serving throughput in a throughput-oriented deployment setting, FP8 and NVFP4 are the most attractive inference formats on this hardware. If instead the deployment is latency-sensitive and operates at small batch sizes, the practical advantage of aggressive low precision becomes much less pronounced, and FP32 or 16-bit execution may remain competitive despite their higher nominal precision.

### 4.5. Ablation of the Mixed-Precision Module Selection Strategy

To justify the selective mixed-precision design adopted throughout this work, we perform an ablation study at the most aggressive training precision, NVFP4, by comparing two configurations: *full quantization*, in which all modules are quantized to NVFP4, and *our mixed-precision design*, in which the initial Conv1 × 1 layer, RoPE, Softmax, and the final MLP classifier head are retained in FP32. Table 6 shows that this design choice is essential for preserving predictive performance.

When all layers are quantized to NVFP4, performance degrades sharply to 84.31% OA, 85.26% AA, and 83.95% κ. By contrast, the proposed mixed-precision design restores performance to 98.25% OA, 98.78% AA, and 98.06% κ, bringing the model within only 0.63 pp of the FP32 baseline in OA. This corresponds to an absolute improvement of 13.94 pp in OA over the full-quantization variant, with similarly large gains in AA and κ. The magnitude of this gap shows that the strong results reported earlier for NVFP4 do not arise from the numerical format alone, but from the combination of low-precision execution in the dominant transformer computations and full-precision retention in numerically sensitive modules.

These findings suggest that the excluded modules are substantially more sensitive to aggressive quantization than the quantized GEMM-dominated layers. The initial Conv1 × 1 projection operates directly on the raw spectral input and therefore likely requires greater numerical fidelity to preserve fine-grained spectral structure before tokenization. The RoPE module encodes relative positional information, where quantization error can distort the spatial relationships needed for local spatial–spectral reasoning. The Softmax operation is also known to be numerically delicate because small perturbations in its inputs can produce amplified changes in the normalized attention distribution. Finally, the classifier head directly maps the learned representation to decision boundaries, making it particularly sensitive to precision loss when classes are separated by subtle spectral differences.

More broadly, this ablation supports the central premise of the proposed mixed-precision strategy: not all modules contribute equally to the numerical robustness of the model. Aggressively quantizing the dominant transformer operations can preserve most of the efficiency benefits of low precision, but only when a small set of sensitive components is protected in FP32. In this compact hyperspectral transformer, selective precision assignment is therefore not merely a refinement for improving accuracy, but a necessary condition for making NVFP4 training viable.

### 4.6. Overall Interpretation and Practical Guidelines

Taken together, the results show that reduced-precision floating-point computation is a viable strategy for compact transformer-based hyperspectral foreign plastic object classification, but its benefits are strongly metric- and workload-dependent. In terms of predictive performance, the mixed-precision FQT design remains highly robust across all tested precisions, with all FQT variants staying within 0.63 percentage points of the FP32 baseline, while consistently outperforming PTQ at matched precisions. BF16 provides the closest accuracy to FP32 mainly because both have the same number of exponent bits, resulting in the same dynamic range, whereas FP8 offers a particularly favorable compromise between accuracy preservation and reduced precision. FP16, on the other hand, despite having a broader dynamic range, still resulted in degraded accuracy compared to FP8 due to using loss scaling as a replacement for a dedicated scaling factor. At the same time, the model-compression results confirm a clear monotonic storage benefit as precision decreases, culminating in 3.69× checkpoint compression under NVFP4. These findings indicate that, for this task, aggressive quantization is feasible when numerically sensitive modules are protected, and quantization is applied selectively rather than uniformly.

The efficiency results reveal a more nuanced systems-level picture. During training, lower precision does not automatically translate into higher throughput for a compact hyperspectral transformer. At the default operating point, FP16 and BF16 provide modest memory savings while preserving FP32-level throughput, whereas FP8 and NVFP4 reduce memory further but incur lower throughput and higher training energy because the workload is too small to fully amortize quantization overhead. Only at sufficiently large batch sizes do FP8 and NVFP4 expose the throughput advantage of native low-precision tensor-core execution, but this is not feasible for training because training batch size is a hyperparameter and increasing the batch size in deep learning models to increase the workload for tensor cores results in accuracy degradation. Inference differs in one important respect: because no optimization is involved, larger batch sizes can be exploited without affecting predictive accuracy. Accordingly, FP8 and especially NVFP4 become the fastest inference formats in throughput-oriented large-batch deployment, while FP32 and 16-bit execution remain competitive at small, latency-sensitive batch sizes. Since FQT- and PTQ-derived models use the same mixed-precision deployment policy, their inference behavior is correspondingly very similar at matched precisions.

These findings can also be viewed in the context of the emerging FQT literature. Recent studies on large transformer and language models have demonstrated that FP8 and FP4 training can preserve higher-precision performance when appropriate scaling and mixed-precision strategies are employed [17,21]. Our results show a similar requirement for numerical care in a substantially smaller and domain-specific HSI transformer, particularly at FP8 and NVFP4. However, unlike the large-model workloads considered in most prior FQT studies, the compact GEMM dimensions of the present model do not consistently saturate low-precision hardware at moderate batch sizes. This explains why reduced numerical precision produces clear storage and accuracy trade-offs but does not automatically translate into higher training throughput. To the best of our knowledge, comparable FQT studies across FP16, BF16, FP8, and FP4 have not previously been reported for transformer-based HSI classification, limiting direct quantitative comparison within the HSI literature.

A final and critical conclusion comes from the ablation study. The strong NVFP4 results are not obtained by simply quantizing the entire network, but by combining low-precision execution in the dominant transformer computations with full-precision retention in a small set of sensitive modules. Quantizing all modules to NVFP4 causes a severe performance collapse, whereas the proposed selective mixed-precision design restores performance to near-FP32 levels. This confirms that the initial spectral projection, RoPE, Softmax, and classifier head are disproportionately sensitive to aggressive quantization and should be preserved at higher precision.

From a practical deployment perspective, the guidelines are therefore as follows. If the primary goal is *maximum accuracy under reduced precision*, BF16-FQT is the most conservative choice, with FP8-FQT offering a strong alternative when slightly more aggressive compression is desired. If *retraining is feasible*, FQT is preferable to PTQ because it consistently delivers better accuracy at matched precision. If *retraining is not feasible*, PTQ remains a practical fallback, particularly at FP16 or BF16, provided that a high-quality representative calibration set is available. If the primary goal is *maximum compression*, NVFP4 is the most effective format, but only under a selective mixed-precision design. If the primary goal is *maximum inference throughput*, FP8 and NVFP4 are attractive only in large-batch serving scenarios; for smaller or latency-sensitive deployments, 16-bit modes may offer a more favorable practical trade-off. Overall, the results suggest that for compact, domain-specific hyperspectral transformers, low precision should be viewed not as a universally beneficial replacement for FP32, but as a workload-aware design choice whose success depends on selective module assignment, scaling strategy, and the intended deployment scenarios.

### 4.7. Limitations

Several limitations of this study should be acknowledged. First, all training hyperparameters were tuned only on the FP32 baseline and then kept fixed across all reduced-precision experiments. This choice was made intentionally to ensure a fair comparison in which performance differences can be attributed to quantization behavior rather than to precision-specific optimization. However, it also means that the reported results may not reflect the best achievable performance for each lower-precision format individually. In particular, some reduced-precision modes might benefit from different learning rates, loss-scaling policies, regularization strengths, or stopping criteria.

Second, the empirical findings are based on a single established state-of-the-art transformer-based model and a single in-house hyperspectral food-safety dataset for poultry FPO classification in the 1000–1700 nm spectral range. Although this controlled design is useful for isolating the effects of numerical precision and quantization strategy, the observed robustness to reduced precision should not be assumed to generalize directly to other transformer architectures, HSI datasets, spectral ranges, sensors, or downstream tasks. Different materials, class structures, spectral characteristics, noise levels, spatial contexts, and environmental conditions may produce different activation distributions and dynamic-range requirements, thereby altering sensitivity to low-precision computation. Future work should therefore evaluate the proposed quantization framework across additional HSI architectures, datasets, spectral ranges, and application domains to establish the broader generalizability of these findings. In addition, the present evaluation uses a labeled pixel-level partition rather than an image-disjoint partition. Consequently, spatially neighboring samples from the same hyperspectral image may occur in different subsets, and their extracted spatial contexts may partially overlap. The reported results therefore characterize a controlled comparison of quantization strategies under a common pixel-level sampling protocol rather than generalization to completely unseen hyperspectral images or material sources. Future work should additionally consider image- and source-disjoint evaluation to assess cross-image and cross-source generalization.

Third, for FP8 and FP4, we adopted specific scaling mechanisms motivated by their strong performance in the LLM literature, namely, tensor-level scaling for FP8 and the NVFP4 tensor-plus-block scaling formulation for FP4. These choices provide a reasonable and practically relevant starting point, but they are not exhaustive. Other scaling mechanisms, including finer-grained or alternative microscaling variants, may behave differently and could prove more suitable for smaller hyperspectral transformer models such as the one studied here.

Fourth, our analysis of workload scaling focused primarily on increasing batch size while keeping the internal tensor dimensions of the selected model unchanged. This was appropriate for isolating the effect of workload on the same backbone under a fair comparison setting. Nevertheless, batch size is only one way to alter the effective GEMM workload. An alternative and complementary approach would be to study a family of models with different widths, depths, or embedding dimensions while holding the batch size fixed. Such experiments could offer further insight into when native FP8 and FP4 hardware support begins to provide consistent end-to-end benefits for hyperspectral transformers.

The reported training and inference efficiency measurements are also specific to the NVIDIA GeForce RTX 5080 Laptop GPU and the associated Blackwell-optimized Transformer Engine software stack used in this study. In particular, the complete precision comparison including native NVFP4 execution requires Blackwell-class hardware support. Therefore, absolute latency, throughput, memory-scaling behavior, and batch-size crossover points should not be interpreted as hardware-independent quantities. These characteristics may vary across GPU architectures, depending on their low-precision tensor core support, memory subsystem, kernel implementations, and software stack. The reported measurements should therefore be interpreted as hardware-specific evidence of the relative scaling behavior observed on the evaluated Blackwell platform, while broader hardware generalization will require evaluation across additional accelerator architectures.

An additional practical limitation concerns FPO visibility and occlusion. This study and its dataset were designed for pixel-level classification of surface-exposed FPOs, with the prepared pieces positioned directly on the fillet surface and visible to the hyperspectral imaging system. Consequently, the reported results do not characterize performance for partially embedded or completely hidden contaminants. Partial occlusion can still be detectable; however, it reduces the number of directly observed FPO pixels and may introduce mixed spectral contributions from both the FPO and surrounding poultry tissue. Complete coverage by tissue may require a mechanical device or robot arm capable of flipping the fillet to inspect both sides, which would necessitate an additional camera downstream before a product rejector or FPO-removal device is used. Future work should therefore investigate controlled levels of partial occlusion, as well as fillet manipulation strategies, to establish the practical detection and classification limits of the proposed framework under more challenging processing conditions.

## 5. Conclusions

This paper presented a systematic investigation of reduced-precision computation for a compact transformer-based hyperspectral classifier in a food-safety application, with a direct comparison of FQT and PTQ across FP16, BF16, FP8, and NVFP4. The results demonstrate that carefully designed mixed-precision FQT can preserve near-FP32 predictive performance, with all evaluated reduced-precision FQT configurations remaining within 0.63 percentage points of the FP32 baseline while consistently outperforming PTQ at matched precisions. Among the evaluated formats, BF16 offered the most robust accuracy preservation, whereas FP8 achieved the best overall balance between predictive performance and computational efficiency. Although NVFP4 provided the greatest reduction in precision and memory footprint, maintaining accuracy required selectively retaining numerically sensitive modules in FP32. From a systems perspective, reduced precision did not inherently improve training throughput for this compact model, whereas FP8 and NVFP4 yielded clear inference benefits under large-batch deployment. Overall, these findings highlight that successful low-precision deployment of compact hyperspectral transformers depends not merely on reducing bit-width, but on adopting appropriate mixed-precision strategies that account for numerical sensitivity, scaling behavior, and deployment workload. The present evaluation is based on FPO pieces of approximately 5×5 mm and therefore does not establish an experimental minimum or maximum detectable object size. From the pixel-level classification perspective, larger FPOs are expected to provide a greater number of spectrally pure target pixels, whereas progressively smaller objects will contain fewer pure pixels and a greater proportion of mixed tissue–FPO boundary pixels. Consequently, the classification of objects below the evaluated size is expected to become increasingly dependent on the spatial resolution of the HSI system. Future work should therefore evaluate controlled FPO dimensions above and below 5 mm to establish the practical size limits of the proposed framework.

## Figures and Tables

**Table 1:** On-disk serialized model sizes per precision for inference under the mixed-precision approach. Compression ratio is relative to the FP32 baseline model checkpoint.

Precision	Retained FP32	Quantized Part	Scale Overhead	Size (MB)	Compression (×)
FP32 (baseline)	1.3624	—	—	1.3624	1.00×
FP16	0.2271	0.5676	0	0.7947	1.71×
BF16	0.2271	0.5676	0	0.7947	1.71×
FP8	0.2271	0.2838	0.0002	0.5111	2.67×
NVFP4	0.2271	0.1419	0.0004	0.3694	3.69×

**Table 2:** Test-set classification accuracy (mean ± std over 10 seeds). FQT trains and evaluates in the target precision; PTQ quantizes a seed-matched FP32 checkpoint and evaluates in the target precision. ${\Delta \mathrm{OA}}_{\mathrm{FP}32}$ is the change relative to the FP32 baseline. Best results per precision between FQT and PTQ flows are **bolded**.

Flow	Precision	OA (%)	AA (%)	κ (%)	${\Delta \mathrm{OA}}_{\mathrm{FP}32}$ (pp)
—	FP32 (baseline)	98.88 ± 0.33	99.21 ± 0.19	98.76 ± 0.37	—
FQT	FP16	**98.33 ± 0.23**	**98.80 ± 0.16**	**98.15 ± 0.26**	**−0.55**
FQT	BF16	**98.60 ± 0.23**	**98.98 ± 0.14**	**98.45 ± 0.25**	**−0.28**
FQT	FP8 ^†^	**98.53 ± 0.17**	**98.97 ± 0.07**	**98.37 ± 0.18**	**−0.35**
FQT	NVFP4 ^‡^	**98.25 ± 0.35**	**98.78 ± 0.21**	**98.06 ± 0.39**	**−0.63**
PTQ	FP16	98.02 ± 0.42	98.59 ± 0.33	97.81 ± 0.47	−0.85
PTQ	BF16	98.08 ± 0.43	98.62 ± 0.35	97.88 ± 0.48	−0.80
PTQ	FP8 ^†^	98.09 ± 0.49	98.62 ± 0.38	97.88 ± 0.54	−0.79
PTQ	NVFP4 ^‡^	97.91 ± 0.26	98.44 ± 0.25	97.69 ± 0.29	−0.97

^†^ FP8 uses tensor-level scaling. ^‡^ NVFP4 uses tensor-level and block-level scaling.

**Table 3:** FQT convergence speed: number of epochs to first reach a given fraction of each format’s own peak validation OA (mean ± std over 10 seeds). Lower = faster convergence.

Precision	Peak Val OA (%)	Ep. to 90%	Ep. to 95%	Ep. to 99%
FP32	99.18 ± 0.07	20 ± 10	43 ± 5	141 ± 18
FP16	98.61 ± 0.06	15 ± 6	52 ± 4	75 ± 1
BF16	98.89 ± 0.09	14 ± 3	34 ± 9	108 ± 26
FP8	98.81 ± 0.16	12 ± 4	33 ± 3	87 ± 9
NVFP4	98.53 ± 0.15	16 ± 4	39 ± 8	96 ± 15

**Table 4:** FQT training efficiency at b=512 (mean ± std over seeds). Throughput speedup and memory reduction are relative to FP32.

Precision	Peak Mem (MB)	Mem Reduction	Throughput (Samp/s)	Speedup	Train Time (h)	Total Energy (Wh)
FP32	306.6	—	23320.1 ± 041.9	—	0.09	2.34 ± 0.00
FP16	272.3	1.13×	23397.0 ± 146.5	1.00×	0.09	2.18 ± 0.01
BF16	272.3	1.13×	23617.5 ± 030.4	1.01×	0.09	2.28 ± 0.01
FP8	247.1	1.24×	14804.1 ± 037.0	0.63×	0.15	3.03 ± 0.02
NVFP4	237.3	1.29×	12778.5 ± 326.8	0.55×	0.16	2.86 ± 0.43

**Table 5:** Peak inference throughput for each (flow, precision) configuration. “Best Batch” is the batch size $b^{*}$ from the sweep $\left\{2^{0},\ldots ,2^{14}\right\}$ that maximizes throughput. Speedup is relative to FP32 peak throughput.

Flow	Precision	Peak Thr. (Samp/s)	Best Batch ($b^{*}$)	Speedup vs. FP32
—	FP32	247,999.6 ± 325.0	16,384	1.00×
FQT	FP16	254,038.5 ± 493.7	16,384	1.02×
FQT	BF16	256,109.1 ± 103.9	16,384	1.03×
FQT	FP8	311,555.1 ± 566.2	16,384	1.26×
FQT	NVFP4	331,544.6 ± 388.1	16,384	1.34×
PTQ	FP16	259,748.8 ± 132.4	16,384	1.05×
PTQ	BF16	259,274.6 ± 372.8	16,384	1.05×
PTQ	FP8	314,523.9 ± 285.2	16,384	1.27×
PTQ	NVFP4	334,164.7 ± 269.7	16,384	1.35×

**Table 6:** Ablation study of the mixed-precision module selection strategy under NVFP4 fully quantized training. “Full quantization” quantizes all modules to NVFP4, whereas “Ours (mixed-precision)” retains the initial Conv1 × 1 layer, RoPE, Softmax, and the final MLP head in FP32. Results are reported as the mean ± standard deviation over 10 seeds.

Configuration	OA (%)	AA (%)	κ (%)
NVFP4—FQ	84.31 ± 0.31	85.26 ± 0.18	83.95 ± 0.53
NVFP4—Ours	98.25 ± 0.35	98.78 ± 0.21	98.06 ± 0.39
FP32—Baseline	98.88 ± 0.33	99.21 ± 0.19	98.76 ± 0.37

## Data Availability

The data presented in this study are available upon request from the corresponding author but are not publicly accessible without authorization from the USDA-ARS. The code will be made public upon publication.

## Abbreviations

The following abbreviations are used in this manuscript:

AA	Average Accuracy
ABS	Acrylonitrile Butadiene Styrene
APE	Absolute Positional Embedding
BN	Batch Normalization
CA	Class Accuracy
CFLA	Center-Focused Linear Attention
CM	Confusion Matrix
CNN	Convolutional Neural Network
DL	Deep Learning
DN	Digital Number
ENVI	Environment Visualizing Imagery
FAB	Fabric
FDA	Food and Drug Administration
FLOP	Floating-Point Operation
FM	Foreign Material
FPO	Foreign Plastic Object
FQT	Fully Quantized Training
HDPE	High-Density Polyethylene
HSI	Hyperspectral Imaging
InGaAs	Indium Gallium Arsenide
Kappa	Cohen’s Kappa Coefficient
LDPE	Low-Density Polyethylene
MAC	Multiply–Accumulate Operation
MLP	Multi-Layer Perceptron
NIR	Near-Infrared
NYL	Nylon
OA	Overall Accuracy
PCA	Principal Component Analysis
PE	Polyethylene
PET	Polyethylene Terephthalate
PLS-DA	Partial Least Squares Discriminant
PP	Polypropylene
PS	Polystyrene
PTFE	Polytetrafluoroethylene
PTQ	Post-Training Quantization
PUR	Polyurethane
PVC	Polyvinyl Chloride
ReLU	Rectified Linear Unit
RGB	Red, Green, Blue
ROI	Region of Interest
RoPE	Rotary Position Embedding
RUB	Rubber
SPA	Successive Projection Algorithm
SVM	Support Vector Machine
TEF	Teflon
USDA	U.S. Department of Agriculture
